# Supplementary material for: Exploration of tetrahedral structures in silicate cathodes using a motif-network scheme
Source: Sci Rep. 2015 Oct 26;5:15555. doi: 10.1038/srep15555 (PMC4620437; doi:10.1038/srep15555)
Supplement: Supplementary Information [file srep15555-s1.pdf]

Supplemental materials to

# Exploration of tetrahedral structures in silicate cathodes using a motif-network scheme

Xin Zhao[1, *], Shunqing Wu[1,2, §], Xiaobao Lv[1,3], Manh Cuong Nguyen[1], Cai-Zhuang Wang[1], Zijing Lin[3], Zi-Zhong Zhu[2], and Kai-Ming Ho[1,4]

[1] Ames Laboratory, US DOE and Department of Physics and Astronomy, Iowa State University, Ames, Iowa 50011, USA

[2] Department of Physics, Xiamen University, Xiamen 361005, China

[3] Department of Physics and Collaborative Innovation Center of Suzhou Nano Science and Technology, University of Science and Technology of China, Hefei 230026, China

[4] International Center for Quantum Design of Functional Materials (ICQD), Hefei National Laboratory for Physical Sciences at the Microscale, University of Science and Technology of China, Hefei 230026, China

[*]email: xzhao@iastate.edu

[§]email: wsq@xmu.edu.cn

## Analysis of deviations from ideal tetrahedral coordination

For an X-centered (X= Mn, Fe, Co or Si) tetrahedron, the distortion parameter is defined as:

$$\Delta = \frac{1}{4}\sum_{i=1}^{4}[(d_i - \langle d\rangle)/\langle d\rangle]^2$$

Where d is the distance between the center atom X and its four O neighbor atoms, <d> is the average distance.

**Table S1.** $Li_2MnSiO_4$. d(X-O) is the average bond length between atom X and O in each structure. Δ(X) is the average distortion parameter of X-centered tetrahedrons in each structure.

| Structure # | Space group | d(Mn-O) (Å) | Δ(Mn) | d(Si-O) (Å) | Δ(Si) |
|---|---|---|---|---|---|
| 1 | 62 | 2.10113 | 4.77E-5 | 1.65663 | 1.25E-5 |
| 2 | 31 | 2.10076 | 4.17E-5 | 1.65676 | 1.03E-5 |
| 3 | 31 | 2.10126 | 3.56E-5 | 1.65714 | 6.9E-6 |
| 4 | 11 | 2.10133 | 1.745E-4 | 1.65638 | 6.5E-6 |
| 5 | 11 | 2.10194 | 2.269E-4 | 1.656 | 1.06E-5 |
| 6 | 14 | 2.09815 | 1.724E-4 | 1.65611 | 5.7E-6 |
| 7 | 33 | 2.08046 | 6.84E-5 | 1.65762 | 1.39E-5 |
| 8 | 7 | 2.08109 | 6.79E-5 | 1.65756 | 1.24E-5 |
| 9 | 7 | 2.08273 | 6.72E-5 | 1.6576 | 1.02E-5 |
| 10 | 14 | 2.08183 | 1.99E-4 | 1.65608 | 1.34E-5 |
| 11 | 1 | 2.08172 | 1.665E-4 | 1.65697 | 1.07E-5 |
| 12 | 4 | 2.08309 | 1.712E-4 | 1.65694 | 8.9E-6 |
| 13 | 7 | 2.08182 | 1.779E-4 | 1.65647 | 6.8E-6 |
| 14 | 33 | 2.08177 | 2.206E-4 | 1.65615 | 1.33E-5 |
| 15 | 1 | 2.08177 | 1.621E-4 | 1.65717 | 8.6E-6 |
| 16 | 7 | 2.08315 | 1.562E-4 | 1.65724 | 6.2E-6 |
| 17 | 62 | 2.10053 | 3.856E-4 | 1.65554 | 1.4E-6 |

| | | | | | |
|---|---|---|---|---|---|
| 18 | 26 | 2.09927 | 1.45E-5 | 1.65743 | 1.1E-6 |
| 19 | 31 | 2.09047 | 6.28E-5 | 1.65864 | 2.23E-5 |
| 20 | 19 | 2.08033 | 2.101E-4 | 1.65697 | 8.6E-6 |
| 21 | 33 | 2.08313 | 2.028E-4 | 1.65707 | 4.9E-6 |
| 22 | 6 | 2.09 | 6.43E-5 | 1.65867 | 2.47E-5 |
| 23 | 11 | 2.10213 | 4.996E-4 | 1.65499 | 5.7E-6 |
| 24 | 31 | 2.08913 | 6.89E-5 | 1.65914 | 2.06E-5 |
| 25 | 14 | 2.07617 | 6.74E-5 | 1.6573 | 6E-6 |
| 26 | 1 | 2.08287 | 3.202E-4 | 1.65644 | 7.1E-6 |
| 27 | 4 | 2.08181 | 3.16E-4 | 1.65643 | 7.4E-6 |
| 28 | 4 | 2.08368 | 3.151E-4 | 1.65656 | 5.2E-6 |
| 29 | 20 | 2.07508 | 1.531E-4 | 1.65546 | 2E-7 |
| 30 | 14 | 2.0847 | 1.476E-4 | 1.65597 | 7E-7 |
| 31 | 33 | 2.0779 | 1.116E-4 | 1.65575 | 7E-6 |
| 32 | 14 | 2.09213 | 2.206E-4 | 1.65627 | 2.97E-5 |
| 33 | 14 | 2.07105 | 1.429E-4 | 1.65528 | 2.3E-6 |

**Table S2.** $Li_2FeSiO_4$. d(X-O) is the average bond length between atom X and O in each structure. Δ(X) is the average distortion parameter of X-centered tetrahedrons in each structure.

| Structure # | Space group | d(Fe-O) (Å) | Δ(Fe) | d(Si-O) (Å) | Δ(Si) |
|---|---|---|---|---|---|
| 1 | 62 | 2.0445 | 2.818E-4 | 1.65608 | 3.5E-6 |
| 2 | 31 | 2.0446 | 2.606E-4 | 1.65626 | 3.9E-6 |
| 3 | 14 | 2.04118 | 3.023E-4 | 1.65517 | 9E-7 |
| 4 | 31 | 2.04456 | 2.233E-4 | 1.65673 | 4.3E-6 |
| 5 | 11 | 2.04455 | 3.74E-4 | 1.65577 | 3E-6 |
| 6 | 11 | 2.04515 | 4.586E-4 | 1.65534 | 2.7E-6 |
| 7 | 19 | 2.02986 | 3.208E-4 | 1.65613 | 1.7E-6 |
| 8 | 1 | 2.02997 | 2.591E-4 | 1.65643 | 3E-6 |
| 9 | 33 | 2.02909 | 1.293E-4 | 1.65686 | 6.9E-6 |
| 10 | 7 | 2.02935 | 1.321E-4 | 1.65684 | 6E-6 |
| 11 | 7 | 2.02898 | 1.294E-4 | 1.65687 | 6.1E-6 |
| 12 | 7 | 2.02933 | 1.327E-4 | 1.65684 | 5.8E-6 |

| | | | | | |
|---|---|---|---|---|---|
| 13 | 33 | 2.03028 | 3.406E-4 | 1.65613 | 2.1E-6 |
| 14 | 7 | 2.03009 | 2.715E-4 | 1.65643 | 3.1E-6 |
| 15 | 7 | 2.03013 | 3.987E-4 | 1.65569 | 4.9E-6 |
| 16 | 14 | 2.02615 | 1.771E-4 | 1.65627 | 6.4E-6 |
| 17 | 1 | 2.03022 | 3.015E-4 | 1.65608 | 4.6E-6 |
| 18 | 62 | 2.04152 | 5.62E-4 | 1.65456 | 3.8E-6 |
| 19 | 4 | 2.03053 | 3.384E-4 | 1.65603 | 5.7E-6 |
| 20 | 1 | 2.03127 | 5.136E-4 | 1.6556 | 2.3E-6 |
| 21 | 4 | 2.03073 | 4.982E-4 | 1.65559 | 2.4E-6 |
| 22 | 14 | 2.03076 | 4.55E-4 | 1.65515 | 1.16E-5 |
| 23 | 4 | 2.03098 | 5.207E-4 | 1.65559 | 2.9E-6 |
| 24 | 11 | 2.05487 | 9.486E-4 | 1.65593 | 2.4E-6 |
| 25 | 33 | 2.03062 | 5.127E-4 | 1.65513 | 1.23E-5 |
| 26 | 31 | 2.03572 | 2.229E-4 | 1.65772 | 1.56E-5 |
| 27 | 6 | 2.03569 | 2.291E-4 | 1.65766 | 1.66E-5 |
| 28 | 4 | 2.02727 | 1.876E-4 | 1.65478 | 4.2E-6 |
| 29 | 11 | 2.0364 | 4.107E-4 | 1.65663 | 9.4E-6 |
| 30 | 7 | 2.0399 | 8.69E-5 | 1.65657 | 2.6E-6 |
| 31 | 11 | 2.04345 | 6.825E-4 | 1.65428 | 8E-7 |
| 32 | 31 | 2.0346 | 2.087E-4 | 1.65816 | 1.64E-5 |
| 33 | 58 | 2.03156 | 2.138E-4 | 1.65811 | 2.5E-5 |
| 34 | 14 | 2.02975 | 3.558E-4 | 1.65681 | 1.3E-5 |
| 35 | 19 | 2.02893 | 3.854E-4 | 1.65672 | 1.23E-5 |
| 36 | 14 | 2.0277 | 2.89E-4 | 1.65785 | 1.48E-5 |
| 37 | 14 | 2.02641 | 2.887E-4 | 1.65768 | 1.43E-5 |
| 38 | 36 | 2.02947 | 2.088E-4 | 1.6589 | 2.39E-5 |
| 39 | 62 | 2.02579 | 5.124E-4 | 1.65651 | 6.6E-6 |
| 40 | 20 | 2.02057 | 1.938E-4 | 1.65463 | 0 |

**Table S3.** $Li_2CoSiO_4$. d(X-O) is the average bond length between atom X and O in each structure. Δ(X) is the average distortion parameter of X-centered tetrahedrons in each structure.

| Structure # | Space group | d(Co-O) (Å) | Δ(Co) | d(Si-O) (Å) | Δ(Si) |
|---|---|---|---|---|---|
| 1 | 7 | 1.99573 | 3.13E-5 | 1.65633 | 2.9E-6 |

| | | | | | |
|---|---|---|---|---|---|
| 2 | 7 | 1.99427 | 2.94E-5 | 1.65636 | 3.4E-6 |
| 3 | 33 | 1.99683 | 8.9E-5 | 1.65384 | 1E-5 |
| 4 | 1 | 1.99478 | 8.56E-5 | 1.65575 | 1.7E-6 |
| 5 | 7 | 1.99376 | 9.35E-5 | 1.65559 | 1E-6 |
| 6 | 1 | 1.99465 | 7.51E-5 | 1.65547 | 2.6E-6 |
| 7 | 4 | 1.99361 | 7.86E-5 | 1.65544 | 2.5E-6 |
| 8 | 7 | 1.99345 | 9.2E-5 | 1.65512 | 1.2E-6 |
| 9 | 31 | 2.01081 | 2.92E-5 | 1.65674 | 2.4E-6 |
| 10 | 14 | 1.99418 | 7.71E-5 | 1.65452 | 6E-6 |
| 11 | 31 | 2.01061 | 2.31E-5 | 1.65656 | 3.6E-6 |
| 12 | 33 | 1.99316 | 8.54E-5 | 1.65444 | 7.2E-6 |
| 13 | 19 | 1.99472 | 1.148E-4 | 1.65541 | 2.4E-6 |
| 14 | 62 | 2.0105 | 1.95E-5 | 1.6563 | 4.2E-6 |
| 15 | 33 | 1.99307 | 1.234E-4 | 1.6554 | 1.1E-6 |
| 16 | 14 | 1.99415 | 7.57E-5 | 1.65567 | 2.4E-6 |
| 17 | 14 | 2.00628 | 6.43E-5 | 1.65564 | 1.2E-6 |
| 18 | 11 | 2.0086 | 5.72E-5 | 1.65582 | 1.6E-6 |
| 19 | 1 | 1.99366 | 1.591E-4 | 1.65489 | 1.5E-6 |
| 20 | 4 | 1.99389 | 1.559E-4 | 1.65487 | 1.8E-6 |
| 21 | 4 | 1.99298 | 1.672E-4 | 1.65487 | 1.1E-6 |
| 22 | 11 | 2.00865 | 6.01E-5 | 1.65566 | 2.9E-6 |
| 23 | 20 | 1.98831 | 1.295E-4 | 1.65391 | 1.7E-6 |
| 24 | 14 | 1.98851 | 1.177E-4 | 1.65406 | 2.4E-6 |
| 25 | 52 | 1.98928 | 1.09E-4 | 1.65425 | 1.8E-6 |
| 26 | 33 | 1.9904 | 8.35E-5 | 1.65467 | 2.9E-6 |
| 27 | 31 | 2.00435 | 4.24E-5 | 1.65805 | 1.05E-5 |
| 28 | 14 | 1.99038 | 6.17E-5 | 1.65468 | 2.6E-6 |
| 29 | 6 | 2.00397 | 4.48E-5 | 1.65805 | 1.19E-5 |
| 30 | 31 | 2.00352 | 5.86E-5 | 1.65852 | 1.09E-5 |

**Table S4.** $Na_2MnSiO_4$. d(X-O) is the average bond length between atom X and O in each structure. Δ(X) is the average distortion parameter of X-centered tetrahedrons in each structure.

| Structure # | Space group | d(Mn-O) (Å) | Δ(Mn) | d(Si-O) (Å) | Δ(Si) |
|---|---|---|---|---|---|

| | | | | | |
|---|---|---|---|---|---|
| 1 | 7 | 2.07798 | 2.97E-5 | 1.65846 | 3.6E-6 |
| 2 | 7 | 2.08432 | 5.99E-5 | 1.65878 | 4.5E-6 |
| 3 | 4 | 2.07824 | 5.13E-5 | 1.6583 | 3.6E-6 |
| 4 | 33 | 2.07723 | 5.04E-5 | 1.65778 | 2.6E-6 |
| 5 | 33 | 2.07743 | 6.42E-5 | 1.658 | 4E-6 |
| 6 | 1 | 2.08335 | 6.71E-5 | 1.65846 | 4.5E-6 |
| 7 | 14 | 2.08512 | 7.72E-5 | 1.65811 | 4.1E-6 |
| 8 | 7 | 2.06932 | 6.17E-5 | 1.65403 | 4E-6 |
| 9 | 20 | 2.07428 | 9.12E-5 | 1.65701 | 1.3E-6 |
| 10 | 4 | 2.07596 | 1.029E-4 | 1.65749 | 3.3E-6 |
| 11 | 1 | 2.07727 | 9.5E-6 | 1.65877 | 2.5E-6 |
| 12 | 14 | 2.08273 | 1.01E-4 | 1.65764 | 2.6E-6 |
| 13 | 14 | 2.08178 | 1.763E-4 | 1.65752 | 8E-7 |
| 14 | 1 | 2.07698 | 5.4E-6 | 1.65877 | 9E-7 |
| 15 | 14 | 2.09341 | 1.381E-4 | 1.65894 | 4.8E-6 |
| 16 | 60 | 2.08654 | 1.159E-4 | 1.65802 | 1.19E-5 |
| 17 | 82 | 2.07478 | 0 | 1.6589 | 0 |
| 18 | 5 | 2.0746 | 0 | 1.65884 | 0 |
| 19 | 7 | 2.08261 | 1.051E-4 | 1.66099 | 3.09E-5 |
| 20 | 1 | 2.08121 | 1.135E-4 | 1.66056 | 2.82E-5 |
| 21 | 2 | 2.09197 | 3.935E-4 | 1.66038 | 8.86E-5 |
| 22 | 7 | 2.08944 | 1.54E-4 | 1.66127 | 3.3E-5 |
| 23 | 7 | 2.08944 | 1.54E-4 | 1.66127 | 3.3E-5 |
| 24 | 14 | 2.09457 | 3.291E-4 | 1.66102 | 9.59E-5 |
| 25 | 14 | 2.10652 | 1.879E-4 | 1.6592 | 2.1E-6 |
| 26 | 56 | 2.08067 | 1.299E-4 | 1.6602 | 3.08E-5 |
| 27 | 14 | 2.08117 | 1.298E-4 | 1.66138 | 3.79E-5 |

**Table S5.** $Na_2FeSiO_4$. d(X-O) is the average bond length between atom X and O in each structure. Δ(X) is the average distortion parameter of X-centered tetrahedrons in each structure.

| Structure # | Space group | d(Fe-O) (Å) | Δ(Fe) | d(Si-O) (Å) | Δ(Si) |
|---|---|---|---|---|---|
| 1 | 7 | 2.02676 | 2.23E-5 | 1.65774 | 7.2E-6 |
| 2 | 33 | 2.02637 | 8.63E-5 | 1.6572 | 8.6E-6 |

| | | | | | |
|---|---|---|---|---|---|
| 3 | 7 | 2.02624 | 7.39E-5 | 1.65693 | 9.2E-6 |
| 4 | 7 | 2.03296 | 5.66E-5 | 1.6581 | 9.5E-6 |
| 5 | 7 | 2.02548 | 5.41E-5 | 1.65677 | 8.7E-6 |
| 6 | 1 | 2.03154 | 5.67E-5 | 1.65751 | 1.08E-5 |
| 7 | 33 | 2.02603 | 1.11E-4 | 1.65683 | 1.57E-5 |
| 8 | 20 | 2.02287 | 1.742E-4 | 1.65626 | 2.9E-6 |
| 9 | 1 | 2.02535 | 1.1E-5 | 1.65787 | 2.9E-6 |
| 10 | 19 | 2.03595 | 6.99E-5 | 1.65774 | 1.83E-5 |
| 11 | 4 | 2.0321 | 4.138E-4 | 1.65687 | 2.2E-6 |
| 12 | 4 | 2.02512 | 1.521E-4 | 1.65626 | 1.4E-5 |
| 13 | 1 | 2.03295 | 6.087E-4 | 1.65785 | 1.2E-5 |
| 14 | 1 | 2.02533 | 8.1E-6 | 1.65791 | 1.1E-6 |
| 15 | 14 | 2.03134 | 2.181E-4 | 1.65679 | 4.6E-6 |
| 16 | 14 | 2.04477 | 3.185E-4 | 1.65828 | 5.6E-6 |
| 17 | 1 | 2.04048 | 6.54E-4 | 1.65821 | 1.73E-5 |
| 18 | 7 | 2.02647 | 7.81E-5 | 1.65949 | 3.18E-5 |
| 19 | 82 | 2.02377 | 0 | 1.65787 | 0 |
| 20 | 56 | 2.02228 | 7.4E-5 | 1.65771 | 2.66E-5 |
| 21 | 2 | 2.04082 | 0.00101 | 1.65967 | 5.35E-5 |
| 22 | 4 | 2.03541 | 8.077E-4 | 1.65796 | 1.24E-5 |
| 23 | 60 | 2.03685 | 2.718E-4 | 1.65744 | 2.19E-5 |
| 24 | 14 | 2.02409 | 6.47E-5 | 1.65934 | 3.24E-5 |
| 25 | 1 | 2.03271 | 1.243E-4 | 1.65929 | 3.04E-5 |
| 26 | 14 | 2.03699 | 8.338E-4 | 1.65903 | 6.4E-5 |
| 27 | 7 | 2.03372 | 1.404E-4 | 1.6598 | 3.37E-5 |
| 28 | 82 | 2.01086 | 0 | 1.65846 | 0 |
| 29 | 20 | 2.03465 | 9.69E-5 | 1.65838 | 5.07E-5 |
| 30 | 5 | 2.01759 | 1.18E-5 | 1.65809 | 1E-7 |
| 31 | 33 | 2.02606 | 9.49E-5 | 1.66003 | 4.66E-5 |
| 32 | 1 | 2.03786 | 8.995E-4 | 1.65797 | 2.01E-5 |
| 33 | 19 | 2.02725 | 8.43E-5 | 1.6591 | 3.04E-5 |
| 34 | 14 | 2.02114 | 1.035E-4 | 1.65848 | 2.31E-5 |

**Table S6.** $Na_2CoSiO_4$. d(X-O) is the average bond length between atom X and O in each structure. Δ(X) is the average distortion parameter of X-centered tetrahedrons in each structure.

| Structure # | Space group | d(Co-O) (Å) | Δ(Co) | d(Si-O) (Å) | Δ(Si) |
|---|---|---|---|---|---|
| 1 | 7 | 1.99222 | 2.72E-5 | 1.65697 | 3.3E-6 |
| 2 | 33 | 1.99009 | 3.9E-5 | 1.65607 | 3.8E-6 |
| 3 | 33 | 1.9911 | 6.85E-5 | 1.65647 | 5.9E-6 |
| 4 | 7 | 1.99943 | 7.45E-5 | 1.65743 | 9.4E-6 |
| 5 | 7 | 1.98604 | 5.11E-5 | 1.65339 | 6.7E-6 |
| 6 | 1 | 1.99803 | 7.97E-5 | 1.65694 | 1.02E-5 |
| 7 | 20 | 1.98891 | 9.63E-5 | 1.65571 | 4.7E-6 |
| 8 | 4 | 1.98857 | 9.65E-5 | 1.65566 | 4.8E-6 |
| 9 | 14 | 1.99902 | 9.1E-5 | 1.65658 | 1.2E-5 |
| 10 | 1 | 1.99022 | 5.3E-6 | 1.65727 | 9E-7 |
| 11 | 4 | 1.99874 | 9.04E-5 | 1.65739 | 8.4E-6 |
| 12 | 82 | 1.98863 | 0 | 1.65761 | 0 |
| 13 | 14 | 1.99541 | 2.208E-4 | 1.65639 | 7.6E-6 |
| 14 | 5 | 1.98812 | 0 | 1.65744 | 0 |
| 15 | 19 | 2.00125 | 1.305E-4 | 1.65707 | 1.86E-5 |
| 16 | 7 | 1.996 | 1.235E-4 | 1.65966 | 3.19E-5 |
| 17 | 1 | 1.99506 | 1.24E-4 | 1.65906 | 3.02E-5 |
| 18 | 2 | 2.00258 | 4.228E-4 | 1.65942 | 1.162E-4 |
| 19 | 19 | 2.00118 | 2.358E-4 | 1.6588 | 7E-5 |
| 20 | 1 | 2.03132 | 5.654E-4 | 1.65777 | 1.12E-5 |
| 21 | 14 | 1.99959 | 2.095E-4 | 1.65859 | 9.46E-5 |
| 22 | 1 | 2.00226 | 1.902E-4 | 1.65948 | 3.77E-5 |
| 23 | 7 | 2.00475 | 2.143E-4 | 1.66008 | 3.86E-5 |
| 24 | 7 | 2.00577 | 2.953E-4 | 1.65827 | 1.26E-5 |
| 25 | 14 | 1.99515 | 1.314E-4 | 1.66007 | 4.19E-5 |
